# *Mycobacterium tuberculosis* Cyclophilin A Uses Novel Signal Sequence for Secretion and Mimics Eukaryotic Cyclophilins for Interaction with Host Protein Repertoire

**DOI:** 10.1371/journal.pone.0088090

**Published:** 2014-02-04

**Authors:** Asani Bhaduri, Richa Misra, Abhijit Maji, Preetida J. Bhetaria, Sonakshi Mishra, Gunjan Arora, Lalit Kumar Singh, Neha Dhasmana, Neha Dubey, Jugsharan Singh Virdi, Yogendra Singh

**Affiliations:** 1 CSIR-Institute of Genomics and Integrative Biology, Delhi, India; 2 Department of Microbiology, University of Delhi, Delhi, India; University of Maryland, United States of America

## Abstract

Cyclophilins are prolyl isomerases with multitude of functions in different cellular processes and pathological conditions. Cyclophilin A (PpiA) of *Mycobacterium tuberculosis* is secreted during infection in intraphagosomal niche. However, our understanding about the evolutionary origin, secretory mechanism or the interactome of *M. tuberculosis* PpiA is limited. This study demonstrates through phylogenetic and structural analyses that PpiA has more proximity to human cyclophilins than the prokaryotic counterparts. We report a unique N-terminal sequence (MADCDSVTNSP) present in pathogenic mycobacterial PpiA and absent in non-pathogenic strains. This sequence stretch was shown to be essential for PpiA secretion. The overexpression of full-length PpiA from *M. tuberculosis* in non-pathogenic *Mycobacterium smegmatis* resulted in PpiA secretion while truncation of the N-terminal stretch obstructed the secretion. In addition, presence of an ESX pathway substrate motif in *M. tuberculosis* PpiA suggested possible involvement of Type VII secretion system. Site-directed mutagenesis of key residues in this motif in full-length PpiA also hindered the secretion in *M. smegmatis*. Bacterial two-hybrid screens with human lung cDNA library as target were utilized to identify interaction partners of PpiA from host repertoire, and a number of substrates with functional representation in iron storage, signal transduction and immune responses were detected. The extensive host interactome coupled with the sequence and structural similarity to human cyclophilins is strongly suggestive of PpiA being deployed by *M. tuberculosis* as an effector mimic against the host cyclophilins.

## Introduction

Pathogenic microbiota, during evolution, have acquired strategic assets like pathogenicity islands, antigenic variation, quorum sensing ability, virulence factors and mimics to combat the eukaryotic host [1]–[3]. *Mycobacterium tuberculosis*, one of the oldest human pathogens, also possesses a complex machinery to subvert host defense mechanisms. Studies on *M. tuberculosis* virulence factors, specialized cell-wall lipids and novel secretory pathways have changed the earlier anthropocentric view of granuloma formation and mycobacterial transmission to a bacteriocentric interpretation [4]–[7]. This is also corroborated by reports on different clinical isolates of *M. tuberculosis* complex where modern lineages have been shown to be more aggressive and instrumental in rapid progression of tuberculosis [8]. A large number of secreted proteins of mycobacteria, some secretory pathways have been identified in recent times and with role of few of these secretory proteins established during infection process; the attention has now refocused on secretome [5], [6], [9], [10].

Cyclophilins are one of the four structurally different groups of peptidyl-prolyl isomerases (the other families being FK506 binding proteins, parvulins and PP2A phosphatase activator), which modulate equilibration of *cis* or *trans* isomers of proline [11], [12]. The cyclophilins belong to an ancient protein family ubiquitous throughout organismal hierarchy [13], [14]. Unlike the eukaryotes, which encode multitude of cyclophilins, most prokaryotes possess a few cyclophilins [15], [16]. The prokaryotic cyclophilins contain a single cyclophilin-like domain (CLD) unlike the large multi-domain eukaryotic counterparts and show a weaker cyclosporin A binding capacity than the eukaryotic cyclophilins [16], [17]. The role of prolyl isomerases has been implicated in diverse vital processes which include, but are not restricted to, protein folding, chaperoning, RNA processing, signal transduction, stress responses and cell death [13], [14], [16]. The functions of these regulators have also been established in various pathological conditions like cancer, Alzheimer's disease, atherosclerosis, diabetes, asthma and in various viral diseases including HIV [14], [18], [19].

*M. tuberculosis* has two cyclophilins, namely cyclophilin A (PpiA) and cyclophilin B (PpiB). The former has been shown to be present in culture filtrates in several studies [10], [20] and is reported to be upregulated in intraphagosomal niche during infection [21]. Although independent groups have partly characterized PpiA with respect to cyclosporin binding or structural moiety [22], [23], no thorough investigation has been made to understand the phylogeny, secretion mechanism and interactome of PpiA.

This study illustrates that *M. tuberculosis* PpiA is a unique cyclophilin with significant sequence and structural similarity to eukaryotic cyclophilins. Comparative sequence analysis of cyclophilins from pathogenic and non-pathogenic mycobacterial species revealed a stretch of N-terminal residues specific to pathogenic mycobacteria. The expression of full-length *M. tuberculosis* PpiA in the non-pathogenic *Mycobacterium smegmatis* resulted in the secretion of PpiA while the secretion of a truncated PpiA (lacking the proposed signal sequence) overexpressed in *M. smegmatis* was almost negligible. Moreover, site directed mutagenesis of a proposed ESX pathway motif (YXXXD/E) in the full-length *M. tuberculosis* PpiA also resulted in loss of secretion in *M. smegmatis* overexpression strain. This suggested a possible involvement of mycobacterial type VII secretion system in PpiA secretion. The bacterial two-hybrid studies using PpiA as bait and human lung cDNA as target revealed a number of interacting partners from human repertoire. The structural overlapping of PpiA with human cyclophilins along with the pathogen specific signal sequence and a wide-ranging host interactome is indicative of a possible mimicry.

## Results and Discussion

### *In silico* analysis of *M. tuberculosis* PpiA

**Figure 1:**
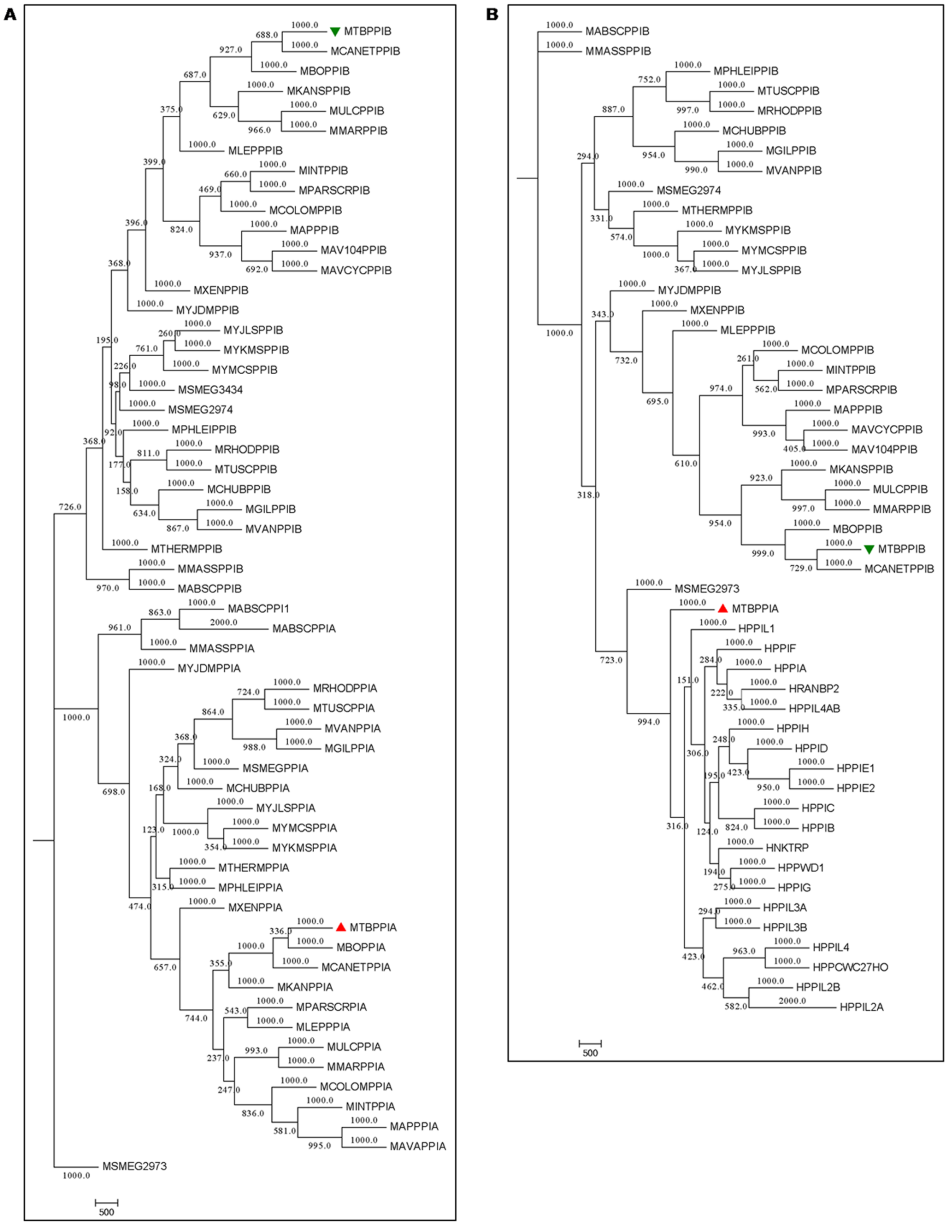
Phylogenetic analysis of *M. tuberculosis* PpiA and PpiB. **Fig. 1A**: The phylogram of mycobacterial cyclophilins revealed two distinct clades of PpiA and PpiB. **Fig. 1B**: *M. tuberculosis* PpiA (MTBPPIA) aligned with human cyclophilins (initials ‘HPP’) instead of clustering with other mycobacterial cyclophilins (initial ‘M’). In both Fig. 1A and B, *M. tuberculosis* PpiA and PpiB are labeled red and green, respectively.

**Figure 2:**
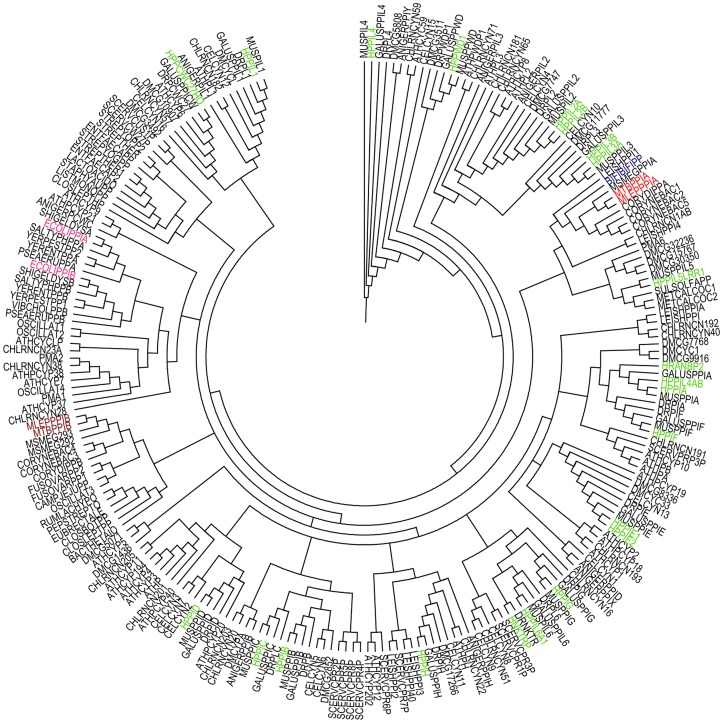
Phylogenetic analysis of cyclophilins. A circular phylogram representation of the cyclophilin sequences collected from various taxa. *M. tuberculosis* PpiA is grouped with eukaryotic and actinobacterial counterparts quite distinct from the prokaryotic clades of cyclophilins. The colored labels are used as follows: human cyclophilins – green, *M. tuberculosis* and *Mycobacterium leprae* PpiA – red, *M. tuberculosis* and *M. leprae* PpiB – brown, *E. coli* cyclophilins – pink and selected gut microbial cyclophilin – dark blue.

**Figure 3:**
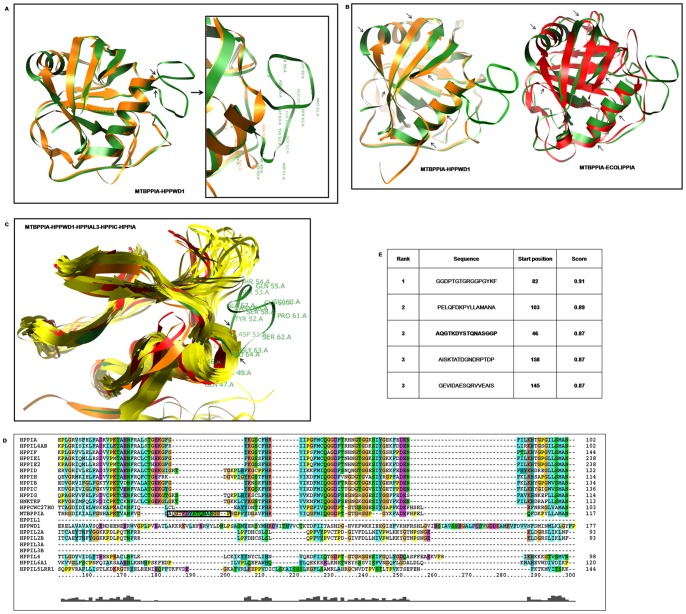
Structural and immunoinformatics analysis of *M. tuberculosis* PpiA. **Fig. 3A**: Structural overlapping of *M. tuberculosis* PpiA (green) with human PPWD1 (yellow) demonstrated almost complete overlap, (inset showing the loop region). **Fig. 3B**: Comparative analysis of structural overlapping between *M. tuberculosis* PpiA (green) with human PPWD1 (yellow) as well as with *E. coli* PpiA (red), respectively, revealed a poor overlap with the prokaryotic cyclophilin. **Fig. 3C**: Structural overlapping of *M. tuberculosis* PpiA (green) and four human cyclophilins (HPPWD1, HPPIAL3, HPPIC, HPPIA) by Chimera program showed almost complete overlap apart from a region of difference (in form of a loop) in *M. tuberculosis* PpiA. **Fig. 3D**: Multiple sequence alignment of the human cyclophilins and *M. tuberculosis* PpiA identified the region of difference ‘AQGTKDYSTQNASGGP’ (inset, black-bordered box). **Fig. 3E**: Immunoinformatics analysis using ABCpred software identified putative epitopic regions in *M. tuberculosis* PpiA.

The growing number of studies depicting the diverse functions of cyclophilins encouraged us to explore mycobacterial cyclophilins, especially PpiA. Multiple sequence alignments and consequent phylogenetic analyses of available mycobacterial cyclophilin protein sequences revealed that both PpiA and PpiB type cyclophilins have conserved sequence patterns and thus form two distinct clusters in mycobacteria (Figure 1A). Since *M. tuberculosis* PpiA was previously shown to bind with cyclosporin A [22], a characteristic unlike of prokaryotic cyclophilins, we next generated a phylogram containing all human cyclophilins, PpiA and several mycobacterial PpiB type cyclophilins. Interestingly, PpiA coaligned with human cyclophilins rather than the mycobacterial PpiB type cyclophilins (Figure 1B). We further aimed to position *M. tuberculosis* PpiA in the organismal hierarchy with a broader phylogenetic analysis. *M. tuberculosis* PpiA and PpiB were aligned with over 200 cyclophilin protein sequences from a wide range of eukaryotes and prokaryotes. In the resultant phylogram, *M. tuberculosis* PpiA was placed incongruently in a clade comprising of eukaryotic and actinobacterial representatives, separated from the prokaryotic clades (Figure 2). Interestingly, *M. tuberculosis* PpiB was also separated from other bacterial cyclophilins and formed a clade of actinobacterial-specific cyclophilins (Figure S1 in File S1). The positioning of *M. tuberculosis* PpiA and PpiB in separate clades than those of prokaryotic cyclophilins suggested that both the mycobacterial cyclophilins belong to an evolutionarily different group from the prokaryotic counterparts. Different outgroupings were then performed to construct separate phylograms for an unbiased analysis. Though PpiB was closer to some of the prokaryotic cyclophilins in few of these resultant trees, in none of these phylogenetic analyses PpiA could be placed with other prokaryotic counterparts (Figure S2 in File S1). The prokaryotic and actinobacterial clades identified in the present work were comparable to a previous *in silico* study on actinobacterial cyclophilins [17]. The apparent differences in other clades could stem from the fact that all 17 human cyclophilins and cyclophilins from various representative organisms were included for a comprehensive analysis in the present work. The clustering of PpiA with actino-eukaryotic group was suggestive of a possible horizontal gene transfer event. Although inter-kingdom gene transfer is shown to be highly prevalent in mycobacteria [24], a direct role of horizontal gene transfer seemed uncertain since flanking region search for the source of *M. tuberculosis ppiA* did not show any match with the human counterparts. The other possibility of *M. tuberculosis* PpiA having eukaryotic resemblance could be due to structural and/or functional mimicry. The ‘Chimera’ program was used for structural alignment studies between *M. tuberculosis* PpiA and human cyclophilins PPWD1, PPIAL3 and PPIC as these were found to possess maximum sequence similarity (46–48%) to *M. tuberculosis* PpiA in the protein BLAST. These were also found to be the closest counterparts of *M. tuberculosis* PpiA in different phylogenetic trees constructed during this work. Human PPIA and *Escherichia coli* PpiA were also selected for overlapping analysis as representatives of eukaryotic and prokaryotic cyclophilins, respectively. *M. tuberculosis* PpiA showed almost complete overlapping with HPPWD1 and other human cyclophilins while a poor overlapping was revealed in structural alignment with *E. coli* PpiA (Figure 3A, B). When a multi-alignment file was created with the cyclophilin domain of PPWD1, PPIAL3, PPIC, PPIA and *M. tuberculosis* PpiA, the overlap was distinctly visible apart from loop insertion in the latter (Figure 3C). A multiple sequence alignment of protein sequence was then performed for PpiA with all human cyclophilins and this loop with a sequence ‘AQGTKDYSTQNASGGP’ was indeed found to be absent in human cyclophilins (Figure 3D). The loop region was also found to be of epitopic nature when immunoinformatics analysis of *M. tuberculosis* PpiA was executed (Figure 3E). This strong structural similarity of a mycobacterial secretory protein to a number of host cyclophilins suggests a possible role of PpiA being deployed as a mimic. Various effector mimic proteins have been characterized from different pathogens like *Salmonella* sp. and *Shigella* sp. [3], [25] and we postulate that *M. tuberculosis* has also engineered a protein (human counterparts of which are regulators of cellular functions) to sabotage the host defense.

### A novel signal sequence and secretion of *M. tuberculosis* PpiA

**Figure 4:**
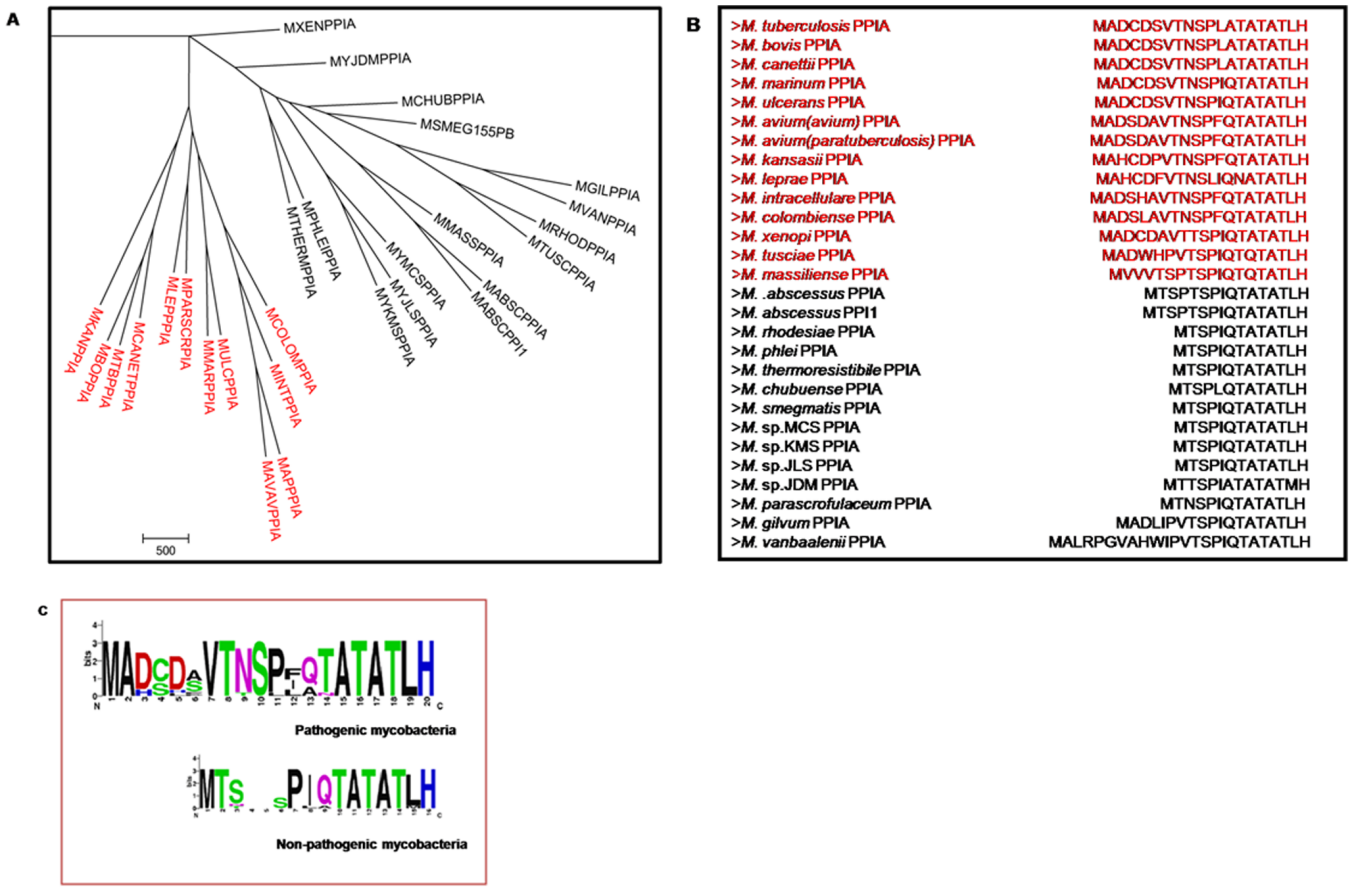
Identification of a novel signal sequence in pathogenic mycobacterial PpiA. **Fig. 4A**: A phylogram of mycobacterial PpiA type cyclophilins revealed the coalescing of pathogenic mycobacteria (red) into a clade, different than that of non-pathogenic mycobacteria (black). **Fig. 4B**: Sequence alignment of the N-terminal stretch revealed that the proposed signal sequence present in pathogenic mycobacterial PpiA was either missing or mismatched in non-pathogenic species. **Fig. 4C**: Sequence logo analysis of the N-terminal stretch of the mycobacterial PpiA type cyclophilins.

**Figure 5:**
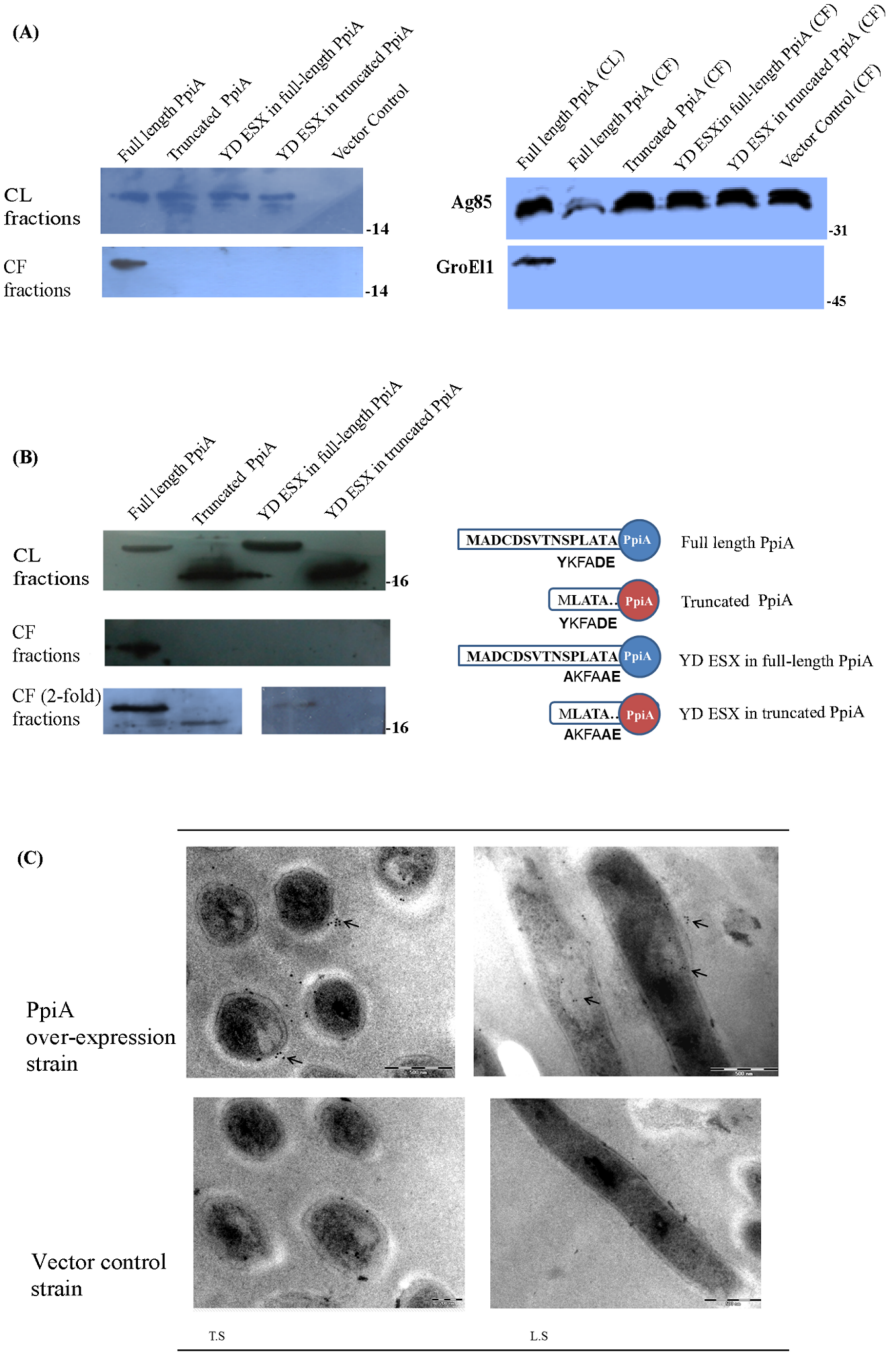
Demonstration of *M. tuberculosis* PpiA secretion and localization. Immunoblotting was performed to show differential secretion pattern of overexpressed full-length, truncated and YD-ESX mutant forms of *M. tuberculosis* PpiA in the culture filtrates of *M. smegmatis*. **Fig. 5A**: Presence of overexpressed full-length, truncated and YD ESX mutant PpiA in the whole cell lysates (CL) of *M. smegmatis* (left upper panel). Secretion of overexpressed full-length PpiA is evident in *M. smegmatis* culture filtrate (CF) while the truncated PpiA without the proposed signal sequence and YD ESX mutant PpiA, though expressed, is not secreted (left lower panel). Expression of Ag85 homolog in all culture filtrates as a control for secretion (right upper panel) and expression of GroEl1 as a cell lysis control and (right lower panel). 12% SDS-PAGE was used in all these blots. **Fig. 5B**: Truncated PpiA and YD-ESX mutant PpiA showed very little amount of secretion even in two-fold culture filtrate concentrate with respect to the full-length PpiA culture filtrate. 16% SDS-PAGE was overrun to distinguish the size difference in these blots. Cell lysate (CL) fractions with two-fold concentrated amount of truncated and YD ESX mutant PpiA, top panel; Equal concentration of all culture filtrates, middle panel; and Culture filtrate fractions with two fold concentrate of truncated and YD ESX mutant PpiA, bottom panel. **Fig. 5C**: Immunoelectron micrograph showing localization of secreted full-length PpiA overexpressed in *M. smegmatis* in comparison with pVV16 vector control in *M. smegmatis*. Arrows indicate the position of PpiA. T.S: Transverse section, L.S: Longitudinal section.

The possibility that *M. tuberculosis* PpiA acts as a virulence factor prompted us to carry out a detailed analysis of the mycobacterial PpiA sequences. A phylogram of available mycobacterial PpiA type cyclophilins separated the pathogenic and non-pathogenic mycobacteria into different clades (Figure 4A). The sequence alignment revealed a stretch of N-terminal amino acids present only in pathogenic mycobacteria (Figure 4B). The first twenty amino acids from pathogenic mycobacteria and the corresponding stretch from non-pathogenic mycobacteria were selected for creation of respective sequence logos (Figure 4C). This alignment showed that approximately 12 amino acids (MADCDSVTNSPL) were conserved among the pathogenic mycobacteria with some variations in D_3_, C_4_, S_6_ and L_12_ residues. In non-pathogenic mycobacteria, this sequence was either absent or replaced by a longer stretch of entirely different amino acids. This N-terminal stretch of amino acids in the cyclophilins of pathogenic mycobacteria appeared like a novel signal sequence. To the best of our knowledge, there is no report of secretion of any non-pathogenic mycobacterial cyclophilin. It was thus presumed that overexpression of PpiA from a pathogenic *Mycobacterium* in a non-pathogenic *Mycobacterium* could result in the secretion of the protein provided the pathway for secretion exists in the bacterium. The overexpression of *M. tuberculosis* PpiA in *M. smegmatis* resulted in secretion of the protein. To prove that this signal sequence was indispensible for proper secretion, a truncated version (lacking the conserved 12 amino acids) of PpiA was created and overexpressed in *M. smegmatis*. The culture filtrate comparison between the strains showed that the full-length form of PpiA was getting secreted while the truncated PpiA was not secreted or secreted below detectable levels (Figure 5A–B). This confirmed that the N-terminal stretch is necessary for secretion and could represent a novel signal sequence. To identify the localization pattern, immunoelectron microscopy was performed with full-length PpiA overexpression strain. The secretion does not seem to be restricted to any specific area, though in longitudinal sections, some patches of secreted proteins were observed near the middle and the terminal portions of the bacteria (Figure 5C).

To identify any components of the secretory pathway involved in PpiA secretion, affinity pull-down experiments were performed with mycobacterial lysates and purified His_6_-tagged *M. tuberculosis* PpiA. We did not find any substrates in the pull down assays with *M. tuberculosis* and *M. smegmatis* whole cell lysates from mycobacterial secretory apparatus. However, these pull-down assays and subsequent mass-spectrometric analysis revealed chaperonin GroEL and elongation factor Tu as interacting partners (Table 1). These interactions confirmed earlier reports depicting interaction between prolyl isomerases and chaperones [15]. The possibility of PpiA secretion through the twin-arginine transport system or general secretory pathways was ruled out since PpiA sequence did not possess the characteristic features of the substrates of these pathways [9], [10]. Nonetheless, a motif ‘Y-XXX-D/E’ responsible for secretion of mycobacterial proteins through ESX system has been identified in a recent study [26] and the PpiA sequence contains a similar motif (‘YKFADE’). To test this hypothesis, we carried out site directed mutagenesis of key residues (Y/A and D/A) in this ESX motif in full-length and truncated PpiA constructs. Upon overexpression, the secretion in the mutant strains was negligible (Figure 5A–B). Although a detailed study is required to come to a conclusion regarding PpiA secretion, our results put forward a possibility that *M. tuberculosis* is using type VII secretion system along with the proposed N-terminal signal sequence to secrete PpiA in the host milieu.

**Table 1:** Mycobacterial proteins interacting with PpiA in pull-down assays.

Protein	H_37_Rv number	Function
GroEL1	Rv3417c	Protein folding, chaperone
Elongation factor Tu	Rv0685	Translation
N-utilization substance protein A (NusA)	Rv2841c	Transcription
Glutamine synthetase (GlnA2)	Rv2222c	Glutamine biosynthesis
Antitoxin VapB8	Rv0664	Antitoxin component

### *M. tuberculosis* PpiA-host interactome

The proposed eukaryotic nature of cyclophilin indicated that once secreted, cyclophilin has the ability to interact with host targets. We performed a bacterial two-hybrid screening (BacterioMatch®) to search for the interaction partners of PpiA. The human lung cDNA library was chosen to accommodate a broad range of genes from different cell clusters in the primary micro-niche of tubercle bacilli (Figure S3 in File S1). The two-hybrid experiments identified a number of PpiA substrates with a wide spectrum of functional diversity (Table 2). These substrates are known to function in iron regulation, immune response, signal transduction, translational apparatus, apoptotic machinery and several other cellular mechanisms. Cyclophilins are known to be involved in intra/extra cellular signaling and so it was not surprising to have some substrates from signal transduction machinery and translational apparatus in PpiA interactome (PTK2, TMEM173, EEF1A1). *M. tuberculosis* PpiA expression is reduced in limited iron conditions [27] and the identification of substrates (ATP6V0E1, FTH1, LCN2) involved in iron uptake, binding and transportation indicates a possible function of PpiA in iron regulation. Some of the interacting partners identified in two-hybrid studies are implicated in receptor signaling and immune response (CD74, IFITM1, LY6E) and suggest a strategy of immune subversion by *M. tuberculosis* through PpiA. Interestingly, various database searches revealed that most of the PpiA substrates were involved in different human disease pathologies including tuberculosis (Table S1 in File S1). The two-hybrid study also detected several immunoglobulin heavy chain variable regions interacting with PpiA. The finding of the epitopic loop in the structural analysis suggested that the peripheral positioning may assist in the interaction of PpiA with the substrates (Figure 3). Together, the results obtained from the library screening and structural analysis suggested PpiA to be a potential antigen for the human host despite the fact that it closely resembled many of the human cyclophilins. This hypothesis was also supported by a recent study depicting PpiA as an immunodominant antigen [28]. Affinity pull-down experiments were also performed with human macrophage and lung epithelial cell lysates to check whether the *ex vivo* interactions could be detected at the *in vitro* level and a few substrates like ferritin, elongation factor, surfactant protein and protein tyrosine kinase were continual with the library screening (Table 3). The identification of the substrate microtubule associated protein tau (MAPT) from the pull-down assay demonstrated the ability of PpiA to act as a molecular mimic as MAPT was previously shown to be a target of prolyl isomerase Pin1 and implicated directly in protection against Alzheimer's disease [29]. We have further validated the interaction of PpiA-EfTu in native PAGE and PpiA-Tau in the pull down experiment using Anti-Tau antibody (Figure S4 in File S1). However, cyclophilin being an extremely interactive protein, further research is required to assess which substrates are encountered and acted upon by *M. tuberculosis* PpiA during the process of infection. The PpiA-host protein-protein interaction landscape, reported in our study, revealed many PpiA substrates which are important either in signaling network or are specific to some selected pathways. Some of these substrates like elongation factor, tyrosine kinase are involved in signal transduction and thus could be termed the ‘hub’ of protein network while proteins like ferritin, surfactant proteins, vacuolar ATPase could represent the ‘bottlenecks’ which are specific and occupy central nodes in the protein-protein interaction networks [30]. A STRING interaction network of the identified substrates revealed interconnectivity through protein-protein interaction profile suggestive of mycobacterial strategy to target specific pathways as well as signaling ‘hub’ (Figure S5 in File S1). In the recent past, cyclophilins have been shown to operate as virulence factors in some pathogenic bacteria and parasitic protozoa [31], [32] and our study also indicates a similar function of *M. tuberculosis* PpiA.

**Table 2:** Identification of interaction partners of PpiA in human lung cDNA repertoire by BacterioMatch® two-hybrid screens.

Gene name	Symbol	Function
Ferritin	**FTH1**	Ferric iron binding
Pulmonary surfactant-associated protein B	**SFTPB**	Surfactant proteins
Pulmonary surfactant-associated protein C	**SFTPC**	Surfactant proteins
Protein tyrosine kinase	**PTK**	Signal transduction
Lipocalin-2	**LCN2**	Iron binding, transporter
Vacuolar ATPase (H+ transporting, lysosomal 9 kDa)	**ATP6V0E1**	Phagosomal maturation, iron uptake
CD74 molecule	**CD74**	MHC classII binding, cytokine response
Elongation factor	**EEF1A1**	Translation
Transmembrane protein 173 (Mediator of IRF3 activation, MPYS,	**TMEM173**	Protein binding, signal transduction
Homo sapiens glucosidase, alpha; neutral AB	**GANAB**	Carbohydrate binding/biosynthesis
Two pore segment channel 1	**TPCN1**	Calcium/votage gated channel activity
KxDL Motif Containing 1	**KXD1**	Protein binding
Interferon Induced Transmembrane Protein 1	**IFITM1**	Immune response, receptor signaling
Lymphocyte Antigen 6 Complex, Locus E	**LY6E**	Receptor linked signaling
Immunoglobulin heavy chain variable regions	**—**	Immune response

**Table 3:** Substrates of PpiA identified from mammalian cell lysates.

Protein	Symbol	Function
Ferritin	**FTL**	Ferric iron binding
Elongation factor EEF1A1	**EEF1A1**	Translation, GTP binding
Pulmonary surfactant associated protein SFTPB/C	**SFTPB/C**	Surfactant proteins
Protein tyrosine kinase PTK2	**PTK**	Signal transduction
Microtubule-associated protein tau	**MAPT, PHF-tau**	Microtubule assembly and stability

## Conclusion

**Figure 6:**
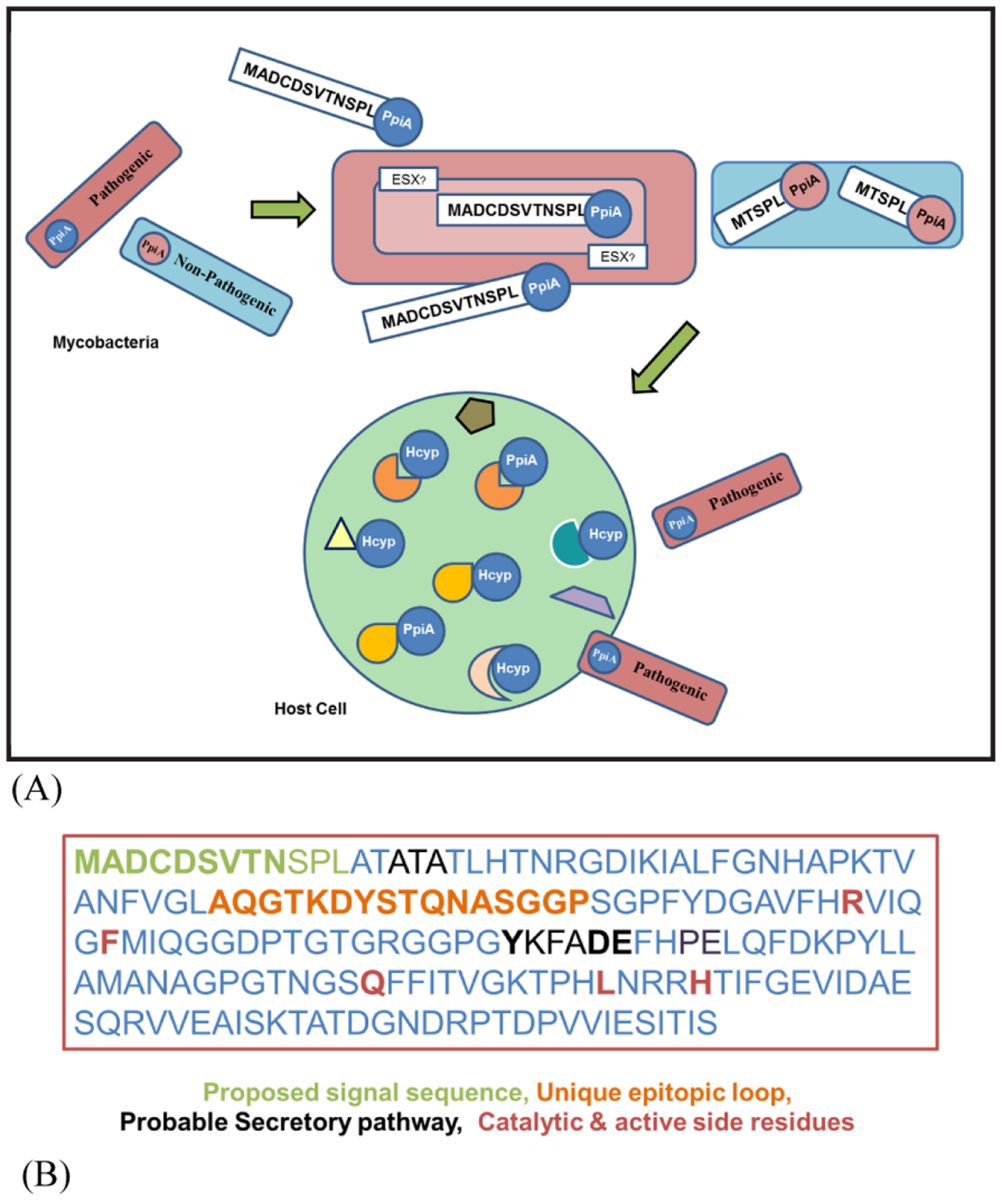
A schematic representation of the proposed mechanism of *M. tuberculosis* PpiA secretion and mimicry along with the important motifs and sequence stretches identified in this study: Fig. 6A: Pathogenic mycobacterial PpiA contains the N-terminal stretch which acts as a signal sequence and aids in PpiA secretion while in non-pathogenic mycobacteria, the truncated PpiA is not secreted or secreted in negligible amount. PpiA might be utilizing the newly identified ESX substrate motif for secretion through mycobacterial type VII secretion system. The phylogenetic and structural similarity of *M. tuberculosis* PpiA to human cyclophilins enables the former to interact with substrates from the host repertoire some of which otherwise could be targeted by the human cyclophilins. The mycobacterial cells are depicted as rectangular shaped rods and the human cell is represented in circular shape. The *M. tuberculosis* PpiA and human cyclophilins are shown in blue circle and named PpiA and Hcyp, respectively. Various other small shapes have been used to represent the protein repertoire within host cell. Fig. 6B: The PpiA amino acid sequence reveals interesting motifs.

This study demonstrates that *M. tuberculosis* PpiA is a eukaryotic-type cyclophilin possibly deployed as a mimic to the human cyclophilins (Figure 6, schematic representation). Our work illustrates the unique positioning of mycobacterial cyclophilins in organismal hierarchy and provides new insights on cyclophilin phylogeny. We have identified a unique signal sequence that is present in pathogenic mycobacterial PpiA and absent in non-pathogenic mycobacterial PpiA. The presence of the signal sequence only in pathogenic mycobacterial PpiA pointed towards the virulence specific function of PpiA. We showed that deleting the signal sequence obstructs the secretion of the protein while the full-length protein when overexpressed in a non-pathogenic *Mycobacterium*, gets secreted. Furthermore, *M. tuberculosis* PpiA also contained a motif sequence similar to those observed in type VII secretion system substrates [26]. Overexpression of mutants of this ESX motif also resulted in loss of secretion in full-length PpiA in *M. smegmatis*, indicating a probable function of ESX pathways in PpiA secretion. Concurrent with the phylogenetic and structural analysis which showed that PpiA is closer to human cyclophilins, we have also identified a significant number of interaction partners of PpiA from the human host, many of which are known to be involved in iron regulation, immune-defense mechanism and signal transduction machinery. This report, therefore, is suggestive of a delicate balance in the host-pathogen interaction wherein *M. tuberculosis* could use the eukaryotic-type cyclophilin to interact and modify certain host proteins while the host immune machinery could try to target the epitopic loop region in PpiA to differentiate this ‘foreign’ cyclophilin from its large repository of ‘self’ cyclophilins.

## Materials and Methods

### *In silico* analysis of cyclophilins

The cyclophilins sequences were retrieved in the FASTA format from NCBI Protein Database (http://www.ncbi.nlm.nih.gov/protein) and by using NCBI protein BLAST (http://blast.ncbi.nlm.nih.gov/Blast.cgi?PAGE=Proteins). *Mycobacterium tuberculosis* PpiA, PpiB and *Homo sapiens* cyclophilins were used as query to retrieve cyclophilin sequences from *Mus musculus*, *Gallus gallus*, *Drosophila melanogaster*, *Danio rerio*, *Leishmania major*, *Caenorhabditis elegans*, *Arapdiopsis thaliana*, *Saccharomyces cerevisiae*, *Aspergillus niger*, *Chlamydomonas reinhardtii*, *Oscillatoria* sp., *Bacteroides thetaiotaomicron*, *Eubacterium rectale*, *Fusobacterium varium*, *Peptostreptococcus anaerobius*, *Streptococcus thermophilus, Bifidobacterium bifidum*, *Lactobacillus acidophilus*, *Ruminococcus lactaris*, *E. coli* K-12, *Bacillus anthracis*, *Campylobacter jejuni*, *Streptococcus pyogenes*, *Staphylococcus aureus*, *Shigella dysenteriae*, *Enterococcus faecalis*, *Yersinia pestis*, *Yersinia enterocolitica*, *Vibrio cholerae*, *Helicobacter pylori*, *Clostridium botulinum*, *Salmonella enterica*, *Pseudomonas aeruginosa*, *Listeria monocytogenes* and available mycobacterial species. Mycobacteriophages were also searched for the presence of cyclophilins, if any, but no homologues were found. Over 280 sequences were used in separate phylogenetic analyses in the present study. All protein sequence accession numbers used in this study have been provided as dataset in File S2 (Dataset). Domain analysis was also carried out to confirm the presence of cyclophilin domain (CLD) in the retrieved sequences by subjecting each sequence to NCBI Conserved Domain Search (http://www.ncbi.nlm.nih.gov/Structure/cdd/wrpsb.cgi). Full-length or selective motif sequences were included for visualizing the phylogenetic tree. Multiple sequence alignments were carried out using CLUSTAL X (2.0.12) [33]. The resulting data were saved as CLUSTAL and PHYLIP format files and alignments were written as postscript files, when needed. The phylogenetic analyses were performed in PHYLIP (ver. 3.69) programs SEQBOOT, PROTDIST, NEIGHBOR, and CONSENSE in the order as described previously [34], [35]. The Jones-Taylor-Thomton amino acid substitution matrix was performed and input order of sequences for phylogenetic analysis was randomized. Phylogenetic tree CONSENSE files obtained from PHYLIP were scrutinized with TREEVIEW (http://taxonomy.zoology.gla.ac.uk/rod/treeview.html) and MEGA (5.0) [36].

ABCpred prediction server was used for immunoinformatics analysis (http://www.imtech.res.in/raghava/abcpred/) to predict B cell epitopes. Structures of the selected sequences were obtained from PDB database, as described previously [13], [23] and sequence alignments were done in CLUSTAL X. Individual or overlapped cyclophilin 3-D structures were viewed in Chimera (http://www.cgl.ucsf.edu/chimera/) [37]. The PDB ids used for structural analyses are as follows: 1W74 (*M. tuberculosis* PpiA), 1LOP (*E. coli* PpiA), 2CPL (Human PPIA), 2A2N (Human PPWD1), 2OK3 (Human PPIL3), 2ESL (PPIC) [13], [23]. Specific regions of interest are shown in Figure 3.

### Cloning of *ppiA* in mycobacterial shuttle vector

The gene fragment coding for *ppiA* (*rv0009*) was PCR amplified from *M. tuberculosis* H_37_Rv genomic DNA using gene specific forward (5′-GCCCGCCCGGGCCATATGGCAGACTGTGATTCC-3′) and reverse primer (5′-GCCGACGTAGCTTCAAGCTTGGAGATGGTGATCGACTCG-3′) and cloned in pVV16 shuttle vector using *Nde*I and *Hind*III restriction enzymes. For the truncated version of *ppiA*, a new primer replaced the forward primer with the sequence (5′-CCGTGACTAACAGCCATATGGCGACCGCTACCGCC-3′). All clones were confirmed by restriction digestion and sequencing.

### Mycobacterial growth conditions and preparation of cell lysate and culture filtrate

*M. tuberculosis* H_37_Rv and *M. smegmatis* MC^2^ 155 were grown at 37°C, in Middlebrook 7H9 broth and Middlebrook 7H10 agar supplemented with 0.5% glycerol, 10% Albumin Dextrose Catalase (ADC) and 0.05% Tween 80 and kanamycin, as required. For preparing the culture filtrate, *M. smegmatis* cultures were grown in 7H9 or Sauton's media in static or mild shaking (50 rpm) condition and after centrifugation the supernatant was filtered through a 0.2-µm filter. The filtrate was concentrated either by passing through Amicon® ultra centrifugal filters or by ammonium sulphate precipitation. Whole cell extracts of *M. tuberculosis* and *M. smegmatis* were prepared as described previously [38]. In brief, cell pellet was harvested by spinning the bacterial culture at 5000 rpm for 10 min and resuspended in phosphate buffered saline (PBS) containing protease inhibitor cocktail (Roche). Zirconium beads were added and bead-beating was performed. The cell debris was sedimented by centrifugation at 13,000 rpm for 30 min and the supernatant was collected and processed as required.

### Generation of antibody for PpiA and immunoblotting

The anti-PpiA antibody was obtained from M/S Indo Biotek, India. In brief, the peptide (‘AQGTKDYSTQNASGGP’-Cys) was selected for conjugation with KLH and antibody raised in rabbit as per standard protocol [39]. Standard protocols of immunoblotting were followed as described before [38], [39]. In brief, concentrated culture filtrate or cell lysate was separated on SDS-PAGE and transferred onto nitrocellulose membrane. After blocking, the blots were incubated with anti-PpiA/anti-His_6_ antibody for 1–2 hr (anti-PpiA dilutions of 1∶10000 in cell lysates and 1∶5000 in culture filtrate; anti-His_6_ dilutions: 1∶15000 in cell lysates and 1∶5000 in culture filtrate). This was followed by washes with PBST and then secondary antibody incubation was performed for 1 hr at room temperature with HRP-conjugated anti-rabbit IgG antibody (Bangalore Genei) at 1∶10,000 dilutions. After three washes, the blots were developed using SuperSignal^R^ West Pico/Femto Chemiluminescent Substrate kit (Pierce, Rockford). Similar results were obtained with anti-PpiA and anti-His_6_ antibodies.

#### Site Directed Mutagenesis

The YD ESX mutants (YFKAD to AFKAA) were generated in full-length and truncated PpiA constructs using the QuikChange XL Site directed Mutagenesis kit (Stratagene) as previously described [39]. The following primers were used for generation of mutant constructs: F.P (5′-CGCGGCGGACCCGGC**GC**CAAGTTCGCCG**C**CGAGTTCCACCCCG-3′) and R.P (5′-CGGGGTGGAACTCG**G**CGGCGAACTTG**GC**GCCGGGTCCGCCGCG-3′).

#### Immunoelectron Microscopy

Immunoelectron microscopy was done as described previously [40]. In brief, the log-phase cultures of *M. smegmatis* were harvested, washed with phosphate buffer (pH 7.2) and fixed in 100 mM sodium cacodylate buffer (pH 7.4) containing 4% paraformaldehyde and 2.5% glutaraldehyde for 24 h. The pellet was osmicated with 0.5% OsO_4_–0.8% K_3_Fe(CN)_6_ followed by 1% tannic acid and stained overnight in 1% uranyl acetate. The cells were gradually dehydrated and embedded in LR White resin and sections were cut using an ultra microtome. Sections on grids were first blocked for 0.5–1 hour followed by washing and overnight incubation at 4°C with rabbit anti-PpiA antibody diluted to 1∶200. The grids were again washed the next day and the bound antibodies were localized by incubation of the sections with secondary gold-conjugated antibody. Grids were washed, stained with 2% aqueous uranyl acetate and observed in a Philips Morgagni 268 microscope.

#### BacterioMatch® two-hybrid screen

BacterioMatch® two-hybrid system vector kit and corresponding *E. coli* competent cells (BacterioMatch® two-hybrid system reporter strain were originally purchased from Stratagene, presently Agilent Technologies, USA) were obtained as a kind gift from Dr. A. Ranganathan (ICGEB, New Delhi) [41]. The BacterioMatch® kit was supplied with bacterial two-hybrid system plasmids, pBT and pTRG, and control plasmids pBT-LGF2 and pTRG-Gal11^p^. BacterioMatch® two-hybrid system was used as described previously [41]. In brief, full-length *ppiA* was PCR amplified from *M. tuberculosis* H_37_Rv genomic DNA using forward (5′-CCGCCCGGAATTCAATGGCAGACTGTGATTCC-3′) and reverse (5′-TGTATTCGAGCCTCGAGCCGACGTAGCTTC-3′) primers containing *Eco*RI and *Xho*I restriction sites as bait in pBT plasmid. Human lung cDNA library available in pTRG plasmid was used as target. The reporter strain *E. coli* XL1 Blue competent cells were co-transformed with equal amounts (250 ng each) of *ppiA*pBT and human lung-cDNApTRG library constructs. The interaction of PpiA expressed from pBT vector to a particular cDNA fragment expressed from pTRG vector results in LacZ expression and growth of blue colonies when plated on X-gal indicator plates containing kanamycin (25 µg/ml), chloramphenicol (30 µg/ml), tetracycline (12.5 µg/ml), X-Gal (80 µg/ml), IPTG (25 µM), and X-Gal inhibitor (200 µM). While pBT and pTRG plasmids alone served as negative controls, pBT-LGF2 and pTRG-Gal11^p^ were used as the positive controls for color scheme for all *ex vivo* interaction studies, as described previously. To avoid false positives, colony PCR was performed for the selected colonies using both *ppiA* specific and vector specific primers to search for the *ppiA* gene and probable cDNA fraction. The PCR confirmed colonies were separated by repeated transformation to divide the plasmids according to their antibiotic resistance and again re-co-transformed to check the presence of coloration in the X-gal indicator plates. The positive cDNA fragments after repeated separation and co-transformation were sequenced and the genes were identified through nucleotide BLAST against human genomic sequences (http://blast.ncbi.nlm.nih.gov/Blast.cgi?PROGRAM=blastn).

#### Purification of His_6_-tagged PpiA protein and pull-down experiments

Recombinant His_6_-tagged *ppiA* was cloned in pProEx-HTc expression vector using (5′-TGGGGGCCCGCCGGATCCTAATGGCAGACTG-3′) forward and (5′-GCCGACGTAGCCTCGAGTCAGGAGATGGTGATCG-3′) reverse primers. Proteins were expressed and purified as described earlier [39]. The purified proteins were analyzed by SDS-PAGE and concentrations were estimated by Bradford assay (Bio-Rad). Affinity pull-down assays were performed with Ni-NTA resin for the His_6_-tagged protein. The resin was washed with binding buffer (300 mM KCl, 30 mM Imidazole, 20 mM Tris (pH-7.4), 1% NP-40, 2 mM EDTA, 2 mM PMSF/PI) and purified His_6_-tagged PpiA protein was slowly added to the resin and allowed to bind for 1 h with Ni-NTA resin at 4°C with gentle shaking. Mycobacterial/mammalian cell lysates were added to the mix and kept for 30 min–1 h at 4°C with gentle shaking. The unbound proteins were collected separately by centrifugation at 3000 rpm for 3 min. The remaining resin was washed thrice with binding buffer. The elution was performed using buffer containing 300 mM KCl, 200 mM Imidazole, 20 mM Tris pH-7.4, 1% NP40, 2 mM PMSF/PI, 2 mM EDTA. The eluted fractions were separated on 12% SDS-PAGE gel and stained with Coomassie blue. The interacting protein bands observed on the gel were cut and processed for MALDI analysis as described earlier (Table S2 in File S1, File S2) [38].

## Supporting Information

File S1 **Figures S1–S5 and Table S1 and S2.** Figure S1. Clade details of *M. tuberculosis* and *E. coli* PpiA and PpiB. The phylogram revealed unique positioning of *M. tuberculosis* PpiA in an actino-eukaryotic clade. The *M. tuberculosis* PpiB was also similarly placed in an actinobacterial clade while both *E. coli* PpiA and PpiB were grouped within a distinct prokaryotic cyclophilin clade. Figure S2. Phylogeny of *M. tuberculosis* PpiA. Several outgroupings was performed in the corresponding CONSENSE tree file of Fig. 2. However, in no resultant tree *M. tuberculosis* PpiA was grouped with prokaryotic clusters. Even in the phylogram shown above, *M. tuberculosis* PpiA is placed in an entirely different clade as that of the prokaryotic cyclophilins. (Colors used as in Fig. 2). Figure S3. Schematic diagram of two hybrid library screen. Fig. S3A: The process involved in BacterioMatch two hybrid library screening. Fig. S3B: The details in the screening of PpiA interaction with inhibitor and positive controls. Figure S4. Interaction of PpiA with *M. tuberculosis* Ef-Tu (Tuf) and human Tau (MAPT) protein. Fig. S4A. Individual lanes showing purified Ef-Tu and PpiA (two right panels) and interaction of Ef-Tu and ppiA showing bands in the top regions (two extreme left panels). Fig. S4B. Blot developed with Anti-Tau antibody (Abcam) showing presence of Tau protein in macrophage (ThP1) cell lysate and PpiA pull-down interaction. Figure S5. STRING interaction network of substrates identified in two-hybrid screening. The display shows the interconnectivity of most of the substrates of *M. tuberculosis* PpiA from the host protein repertoire. Table S1. The host substrates of *M. tuberculosis* PpiA has been implicated in various pathological conditions and the expression level of PpiA interacting partners has been shown to be altered in different diseases including tuberculosis. (Source: literature search, databases like Gene expression Atlas, GeneCards, RefGene, OMIM, BioGRID, STRING). Table S2. Cell lines in which lysates pull-down interaction between purified *M. tuberculosis* PpiA and human substrates were identified.
(DOCX)

File S2 **Protein accession numbers and nomenclature of the cyclophilin sequences retrieved and used in this study.**
(XLSX)
