# Supplementary material for: Mycobacterium tuberculosis Cyclophilin A Uses Novel Signal Sequence for Secretion and Mimics Eukaryotic Cyclophilins for Interaction with Host Protein Repertoire
Source: PLoS One. 2014 Feb 4;9(2):e88090. doi: 10.1371/journal.pone.0088090 (PMC3913756; doi:10.1371/journal.pone.0088090)
Supplement: File S1 — Figures S1–S5 and Table S1 and S2. Figure S1. Clade details of M. tuberculosis and E. coli PpiA and PpiB. The phylogram revealed unique positioning of M. tuberculosis PpiA in an actino-eukaryotic clade. The M. tuberculosis PpiB was also similarly placed in an actinobacterial clade while both E. coli PpiA and PpiB were grouped within a distinct prokaryotic cyclophilin clade. Figure S2. Phylogeny of M. tuberculosis PpiA. Several outgroupings was performed in the corresponding CONSENSE tree file of Fig. 2. However, in no resultant tree M. tuberculosis PpiA was grouped with prokaryotic clusters. Even in the phylogram shown above, M. tuberculosis PpiA is placed in an entirely different clade as that of the prokaryotic cyclophilins. (Colors used as in Fig. 2). Figure S3. Schematic diagram of two hybrid library screen. Fig. S3A: The process involved in BacterioMatch two hybrid library screening. Fig. S3B: The details in the screening of PpiA interaction with inhibitor and positive controls. Figure S4. Interaction of PpiA with M. tuberculosis Ef-Tu (Tuf) and human Tau (MAPT) protein. Fig. S4A. Individual lanes showing purified Ef-Tu and PpiA (two right panels) and interaction of Ef-Tu and ppiA showing bands in the top regions (two extreme left panels). Fig. S4B. Blot developed with Anti-Tau antibody (Abcam) showing presence of Tau protein in macrophage (ThP1) cell lysate and PpiA pull-down interaction. Figure S5. STRING interaction network of substrates identified in two-hybrid screening. The display shows the interconnectivity of most of the substrates of M. tuberculosis PpiA from the host protein repertoire. Table S1. The host substrates of M. tuberculosis PpiA has been implicated in various pathological conditions and the expression level of PpiA interacting partners has been shown to be altered in different diseases including tuberculosis. (Source: literature search, databases like Gene expression Atlas, GeneCards, RefGene, OMIM, BioGRID, STRING). Table S2. Cell lines i [file pone.0088090.s001.docx]

**Supplementary File 1 (S1)**

***Mycobacterium tuberculosis* cyclophilin A uses novel signal sequence for secretion and mimics eukaryotic cyclophilins for interaction with host protein repertoire**

Asani Bhaduri, Richa Misra, Abhijit Maji, Preetida J. Bhetaria, Sonakshi Mishra, Gunjan Arora, Lalit Kumar Singh, Neha Dhasmana, Neha Dubey, Jugsharan Singh Virdi, Yogendra Singh


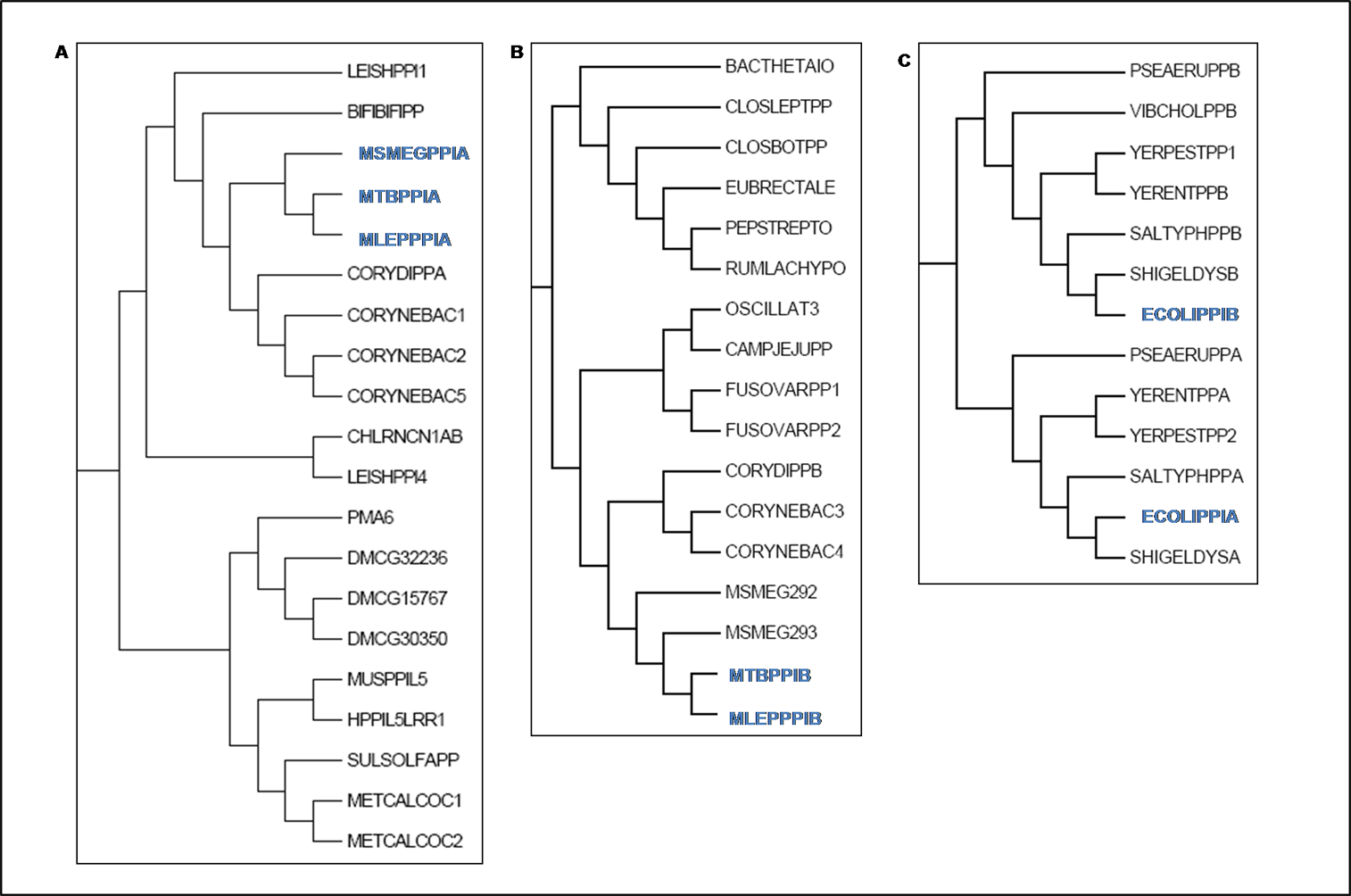


**Figure S1**. **Clade details of *M. tuberculosis* and *E. coli* PpiA and PpiB.** The phylogram revealed unique positioning of *M. tuberculosis* PpiA in an actino-eukaryotic clade. The *M. tuberculosis* PpiB was also similarly placed in an actinobacterial clade while both *E. coli* PpiA and PpiB were grouped within a distinct prokaryotic cyclophilin clade.


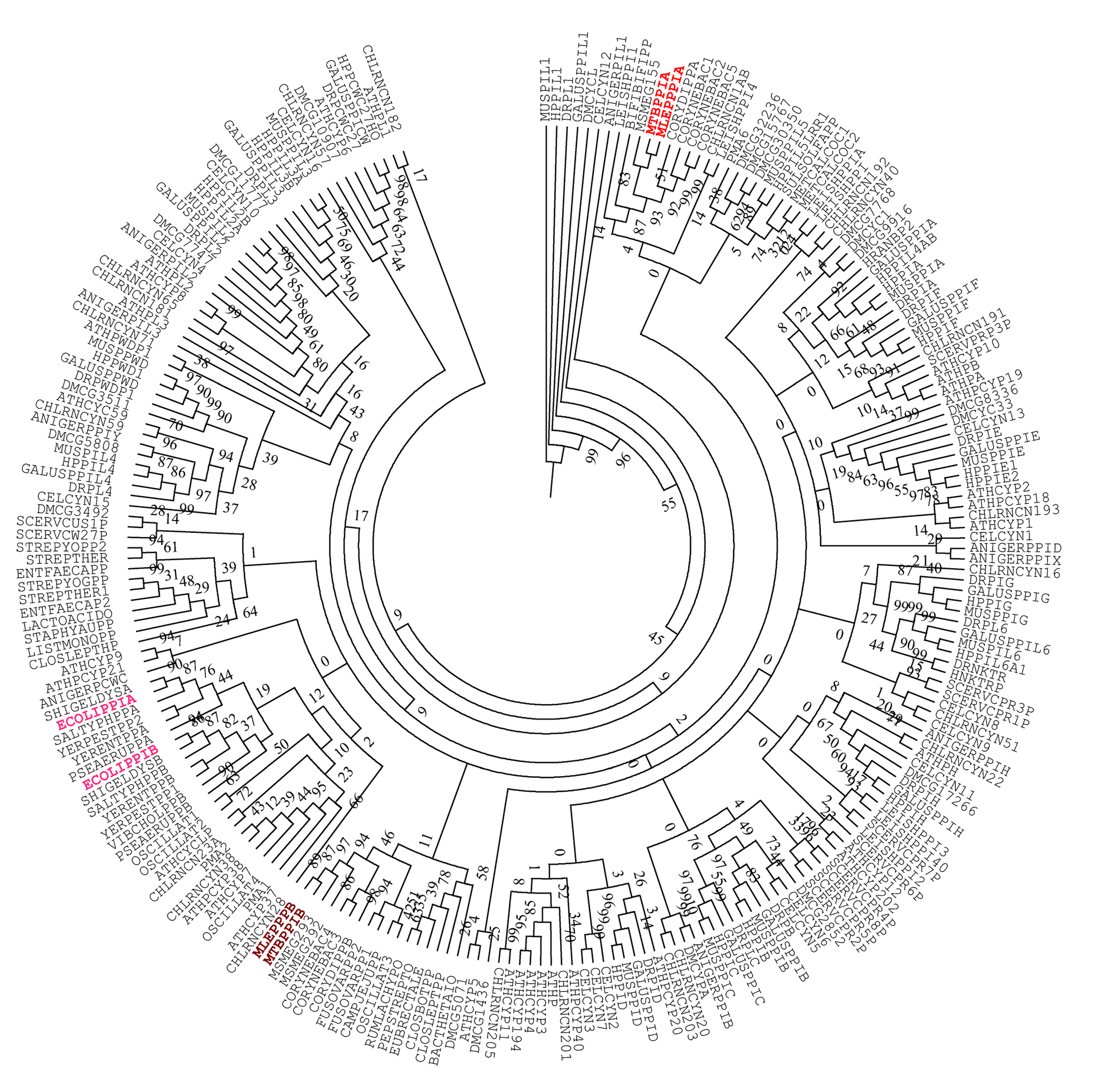


**Figure S2**. **Phylogeny of *M*. *tuberculosis* PpiA.** Several outgroupings was performed in the corresponding CONSENSE tree file of Fig. 2. However, in no resultant tree *M*. *tuberculosis* PpiA was grouped with prokaryotic clusters. Even in the phylogram shown above, *M*. *tuberculosis* PpiA is placed in an entirely different clade as that of the prokaryotic cyclophilins. (Colors used as in Fig. 2).

**A
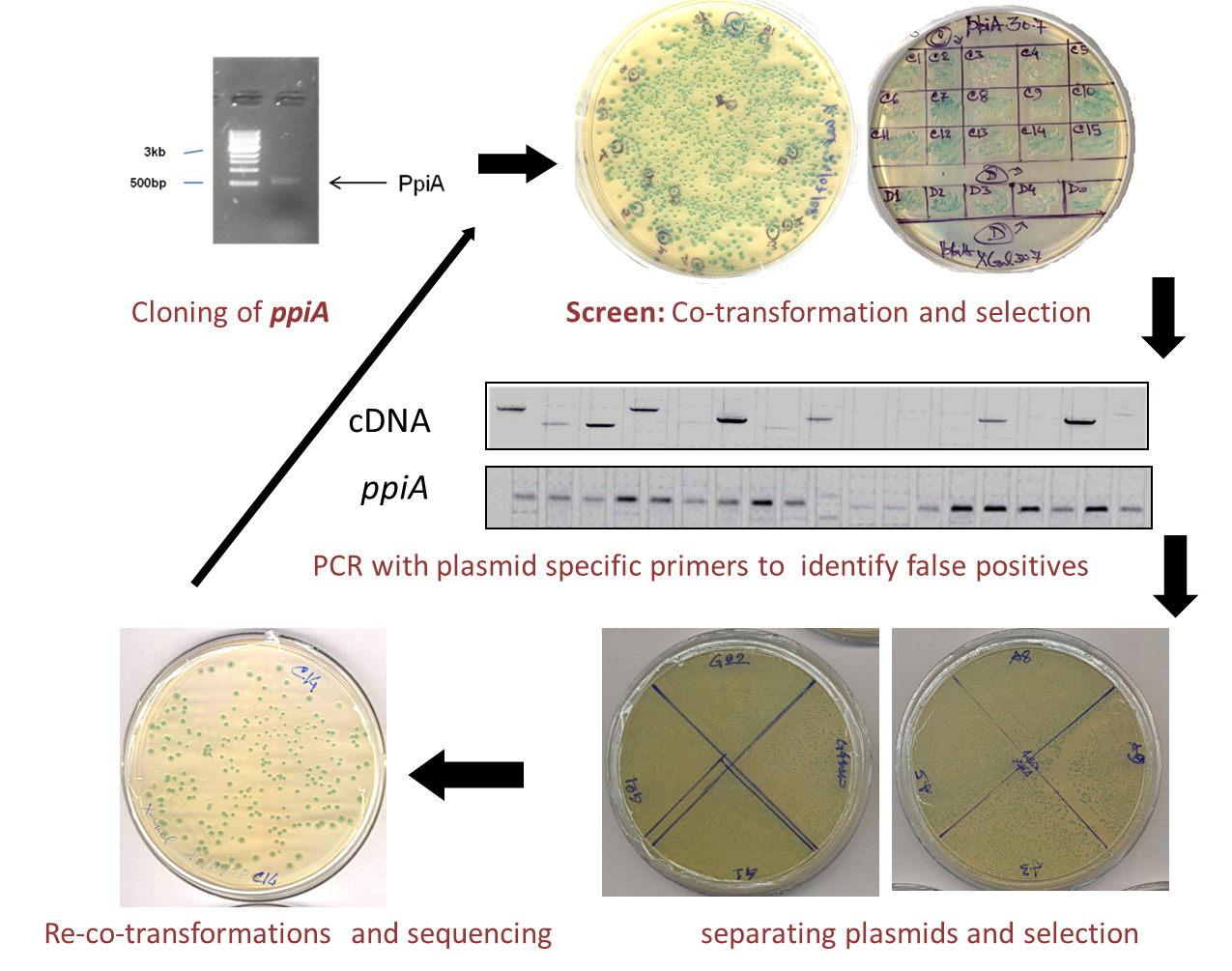
**

**B
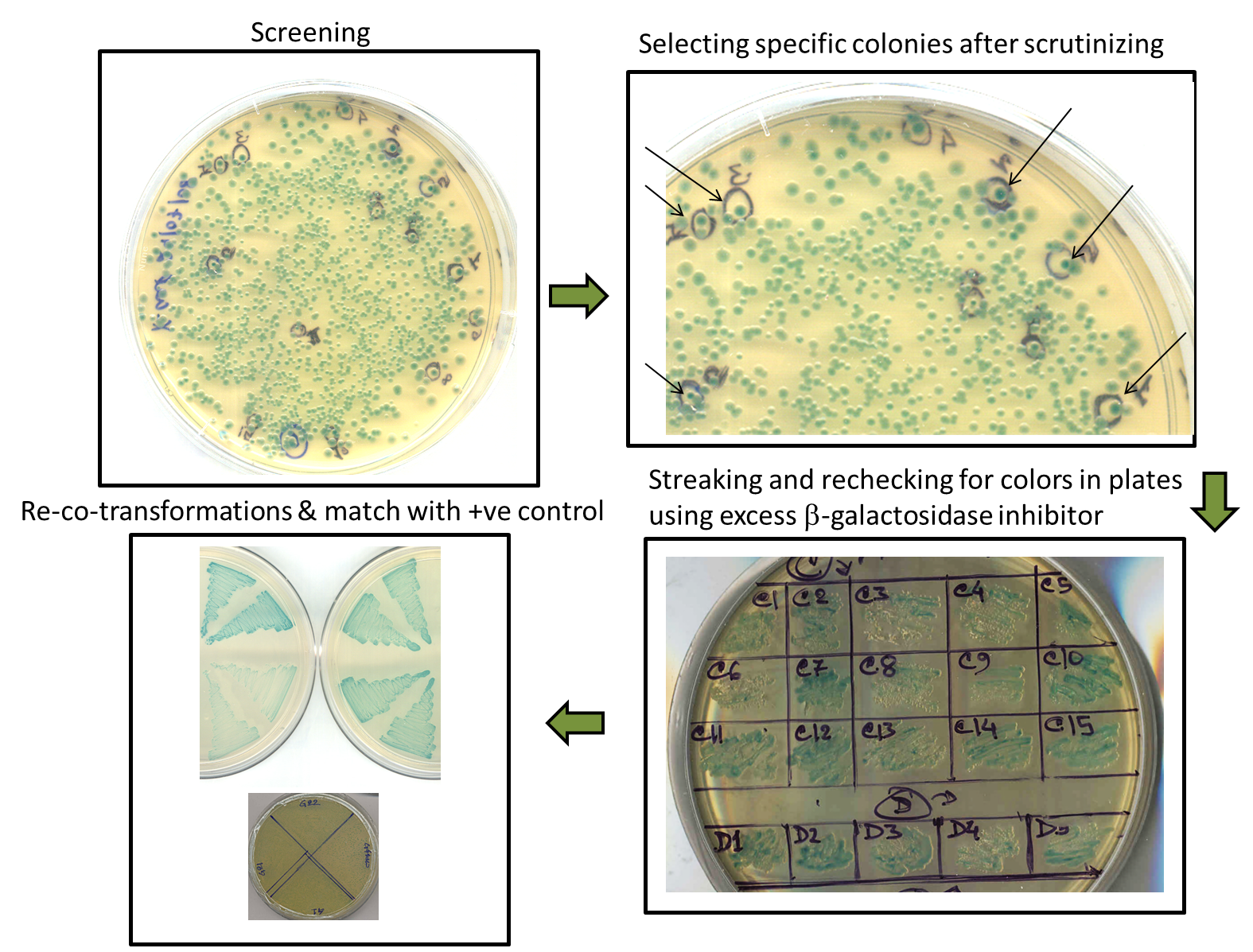
**

**Figure S3. Schematic diagram of two hybrid library screen. Fig. S3A:** The process involved in BacterioMatch two hybrid library screening. **Fig. S3B:** The details in the screening of PpiA interaction with inhibitor and positive controls.

A B

**Figure S4: Interaction of PpiA with *M. tuberculosis* Ef-Tu (Tuf) and human Tau (MAPT) protein. Fig. S4A. Individual lanes showing purified Ef-Tu and PpiA (two right panels) and interaction of Ef-Tu and ppiA showing bands in the top regions (two extreme left panels). Fig. S4B. Blot developed with Anti-Tau antibody (Abcam) showing presence of Tau protein in macrophage (ThP1) cell lysate and PpiA pull-down interaction.**


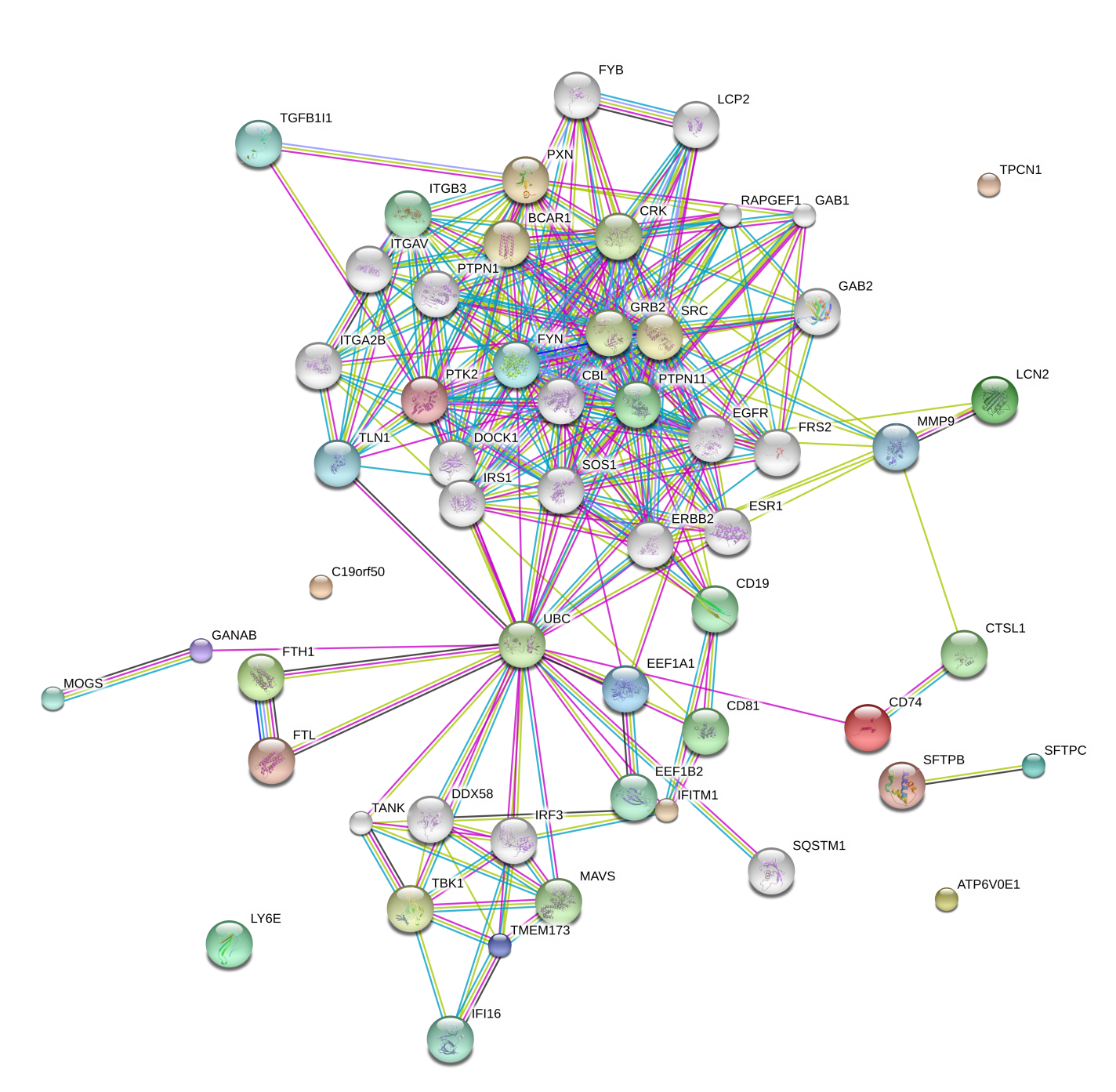


**Figure S5. STRING interaction network of substrates identified in two-hybrid screening.** The display shows the interconnectivity of most of the substrates of *M. tuberculosis* PpiA from the host protein repertoire.

**Table S1. The host substrates of *M. tuberculosis* PpiA has been implicated in various pathological conditions and the expression level of PpiA interacting partners has been shown to be altered in different diseases including tuberculosis.** (Source: literature search, databases like Gene expression Atlas, GeneCards, RefGene, OMIM, BioGRID, STRING).

| **Symbol** | **Diseases** | **Tuberculosis** |
| --- | --- | --- |
| **FTH1** | Lung squamous cell carcinoma | Downregulated in BCG challenge |
| **SFTPB** | Lung injury, tumor | - |
| **SFTPC** | Lung injury, tumor | - |
| **PTK** | Hepatocellular carcinoma | *M. tuberculosis* induces PTK activity |
| **LCN2** | Inflammation, renal injury, | Inhibition of *M. tuberculosis* |
| **ATP6V0E1** | Cancer | Involved in phagosomal maturation |
| **CD74** | AIDS, Viral disease, Diabetes | - |
| **EEF1A1** | Prostate cancer | Expression altered in BCG challenge |
| **TMEM173** | Viral diseases | Innate immune signalling |
| **GANAB** | AIDS, Head/neck cancer | - |
| **TPCN1** | Liver disease | Overexpression in leprosy |
| **KXD1** | Autophagy, gastric cancer | Involved in lysosomal biogenesis |
| **IFITM1** | Viral infection, Cancer | - |
| **LY6E** | SLE | - |

**Table S2. Cell lines in which lysates pull-down interaction between purified *M. tuberculosis* PpiA and human substrates were identified.**

| **Protein** | **Cell Lysate for pull down** |
| --- | --- |
| Ferritin | (THP1) |
| Elongation factor EEF1A1 | (A549) |
| Pulmonary surfactant associated protein SFTPC | (A549) |
| Protein tyrosine kinase PTK2 | (A549) |
| Microtubule-associated protein tau | (THP1) |
